# Reducing eukaryotic viruses while preserving the phageome to achieve a safer virome for fecal virome transplantation

**DOI:** 10.1016/j.psj.2026.107227

**Published:** 2026-06-02

**Authors:** Huimin Li, Colin Buttimer, Yujuan Zeng, Xinglong Li, Yimin Jia, Maoda Pang, Yue Li, Hui Zhang, Yan Zhou, Ran Wang, Hongduo Bao

**Affiliations:** a College of Veterinary Medicine, Nanjing Agricultural University, Nanjing 210095, China; b Jiangsu Key Laboratory of Food Quality and Safety-State Key Laboratory Cultivation Base of MOST, Institute of Food Safety and Nutrition, Jiangsu Academy of Agricultural Sciences, Nanjing 210014, China; c APC Microbiome Institute, University College Cork, Cork T12 YT20, Ireland; d College of Animal Science and Technology, Nanjing Agricultural University, Nanjing 210095, China; e College of Life Sciences and Food Engineering, Huai’an University of Technology, Huai′an 223003, China

**Keywords:** Fecal virome transplantation, Bacteriophages, Eukaryotic viruses, Solvent/detergent

## Abstract

The virome plays a significant role in maintaining the gut health of the host. Fecal virome transplantation (FVT), a burgeoning therapeutic strategy, holds promise in regulating intestinal microecology and treating associated diseases. However, the presence of eukaryotic viruses in FVT poses potential risks, which may compromise its safety and efficacy. This study leverages the key distinction between bacteriophages and eukaryotic viruses—namely, the presence or absence of an envelope—to reduce the burden of eukaryotic viruses in samples via solvent/detergent (S/D) treatment, while preserving the biologically active phageome. To this end, fecal samples were collected from healthy AA broilers, and the virome was isolated and subjected to S/D treatment. Subsequent DNA virome sequencing analysis was conducted to evaluate the impact of this treatment. The results indicated that S/D treatment tended to decrease the relative abundance of eukaryotic viral families (e.g., *Adenoviridae, Parvoviridae*), while bacteriophages remained the dominant viral component. α-diversity analysis revealed no significant differences in overall viral diversity post-treatment. However, β-diversity analysis indicated shifts in viral community composition. Further differential virus analysis revealed a significant increase in the relative abundance of specific bacteriophages following treatment. Finally, functional analysis of the virome revealed a diverse array of genes involved in DNA metabolism and host cell wall remodeling. Notably, the primary functional distinction between FVT0 and FVT1 was the attenuated response to eukaryotic viruses in FVT1. Collectively, this study suggests that S/D treatment may reduce the abundance of eukaryotic viruses in FVT, thereby enhancing clinical safety. These findings provide a theoretical basis and methodological support for the safe application of FVT in the treatment of animal diseases, paving the way for more secure and effective viral therapies.

## Introduction

The gut microbiota constitutes a complex micro-ecosystem comprising bacteria, fungi, viruses, archaea, and other microbial entities (Martinez et al., 2022). Homeostasis of this microbial community is crucial for host health, as it contributes to efficient digestion and nutrient absorption, regulates metabolic and immune functions, and provides resistance against pathogenic invasion (Malik et al., 2025). Consequently, the restoration of a disrupted microbiota has become a major research focus. Among various strategies, fecal microbiota transplantation (FMT) is a promising therapeutic approach for diseases associated with dysbiosis. It aims to restore a healthy microbial composition and function by transferring fecal material from a healthy donor to a recipient (Ng et al., 2024). For instance, studies have demonstrated that FMT can effectively restore gut microbiota composition in DSS-induced colitis models, returning the relative abundances of phyla such as *Bacteroidetes* and *Proteobacteria* to levels comparable to those of healthy controls (Zhang et al., 2020).

Despite its efficacy and favorable short-term safety profile, FMT faces significant challenges, including a lack of standardization and potentially incalculable long-term risks (Yu et al., 2023). To address issues of availability and safety, filtered fecal transplant preparations, known as fecal virome transplantation (FVT), have been developed. FVT has shown therapeutic potential in models of metabolic syndrome, type 2 diabetes (T2D), and obesity (Rasmussen et al., 2020; Manrique et al., 2021). FVT involves sterile filtration of FMT material to remove bacteria and fungi, thereby mitigating risks such as bacterial translocation during viral component transfer (the gut virome). The gut virome is predominantly composed of bacteriophages (phages), approximately 97.7%, which are the primary focus of FVT research due to their potential to modulate bacterial populations and influence host health (Dalmasso et al., 2014; Wu et al., 2023). While studies have characterized the viral fraction in healthy populations and various disease states, the majority of the mammalian gut viromes are uncharacterized due to a lack of comprehensive phage sequence databases (Carding et al., 2017; Shkoporov et al., 2019; Zuo et al., 2019). This has led to its description as “viral dark matter”. However, FVT can also contain eukaryotic viruses and archaeal viruses (Spencer et al., 2022). While many of these may be commensal or harmless, their potential role in disease pathogenesis remains a safety concern that warrants investigation.

A key structural difference between these viral groups can be exploited to enhance FVT safety: most eukaryotic viruses are enveloped, whereas the vast majority of phages are non-enveloped (Adriaenssens et al., 2020; Roux et al., 2023). Enveloped viruses are susceptible to inactivation by solvents and detergents (S/D). Therefore, to selectively reduce the load of eukaryotic viruses while preserving the bioactive phage community, we treated an FVT preparation derived from healthy broiler chickens with an S/D protocol. The feasibility of this method for achieving selective viral inactivation was assessed by comparing DNA virome sequencing profiles of samples taken before and after S/D treatment.

## Materials and methods

### Fecal virome preparation

Three fecal samples were collected. Each of these samples was a composite of feces gathered from 20 healthy 42 - day - old AA broilers. Subsequently, these three fecal samples were individually homogenized in sterile SM buffer. The resulting suspension was centrifuged (4,500 × g, 30 min, 4 °C) to pellet debris, and the supernatant was sequentially filtered through a 0.45 μm membrane to remove bacteria and larger particles. Viral particles were subsequently concentrated via ultrafiltration using a Centriprep Ultrafiltration YM-30 K device (Millipore). The 30 kDa filter membrane was aseptically excised and incubated with the fecal virome at 4 °C overnight to facilitate viral particle diffusion. Subsequently, sterilize the sample by filtration through a 0.22 μm membrane to obtain the fecal virome (termed FVT0).

### Solvent/detergent-treated fecal virome

Following the methodology established by Mao et al., 2024, each half-aliquot of the fecal virome suspension was treated with 1% (w/v) tri (n‑butyl) phosphate (TnBP) and 1% (w/v) Triton X-100 at 30 °C for 4 h. According to the method of Trescec et al., 1999, Amberlite XAD-7 resin (Thermo Scientific) was used to remove TNBP and Triton X-100. The resin column was first equilibrated with 0.01 M phosphate buffer (pH 7.0, containing Na_2_HPO_4_ and NaH_2_PO_4_) supplemented with 0.5 M NaCl until the optical density at 280 nm (OD_280_) of the eluate fell below 0.02. Subsequently, the processed fecal virome suspension was loaded onto the column, and the eluate was monitored by measuring OD_280_ until it also fell below 0.02. To release any potential absorbed viral particles from the resin, a 0.01 M phosphate buffer containing 1 M NaCl was used for elution. The obtained mixture was then concentrated using the Ultracel YM-30 K centrifugal filter device, yielding the final products (termed FVT1). The concentrated product was then stored at −80 °C until further use. To ensure the reliability and reproducibility of the results, this entire experiment was independently repeated three times.

### DNA virome sequencing and analysis

Three samples were processed, namely FVT-0, FVT-10 and FVT-20. Post processing were termed FVT-1, FVT-11 and FVT-21. For each of the three sample pairs (FVT-0 and FVT-1, FVT-10 and FVT-11, FVT-20 and FVT-21), 3 mL aliquots were collected both prior to and following solvent/detergent treatment. Genomic DNA was then extracted from these aliquots using a phage DNA isolation kit (Norgen Biotek, Canada) according to the manufacturer's instructions. Subsequently, the extracted DNA samples were sent to Shanghai 3D Biomedicine Science and Technology Co., Ltd. (Shanghai, China) for sequencing. After the sequencing process was completed, the raw data were sent to Chengdu Phagetimes Biotech Co., Ltd (Chengdu, China) for comprehensive DNA virome analysis.

**Fig. 1:**
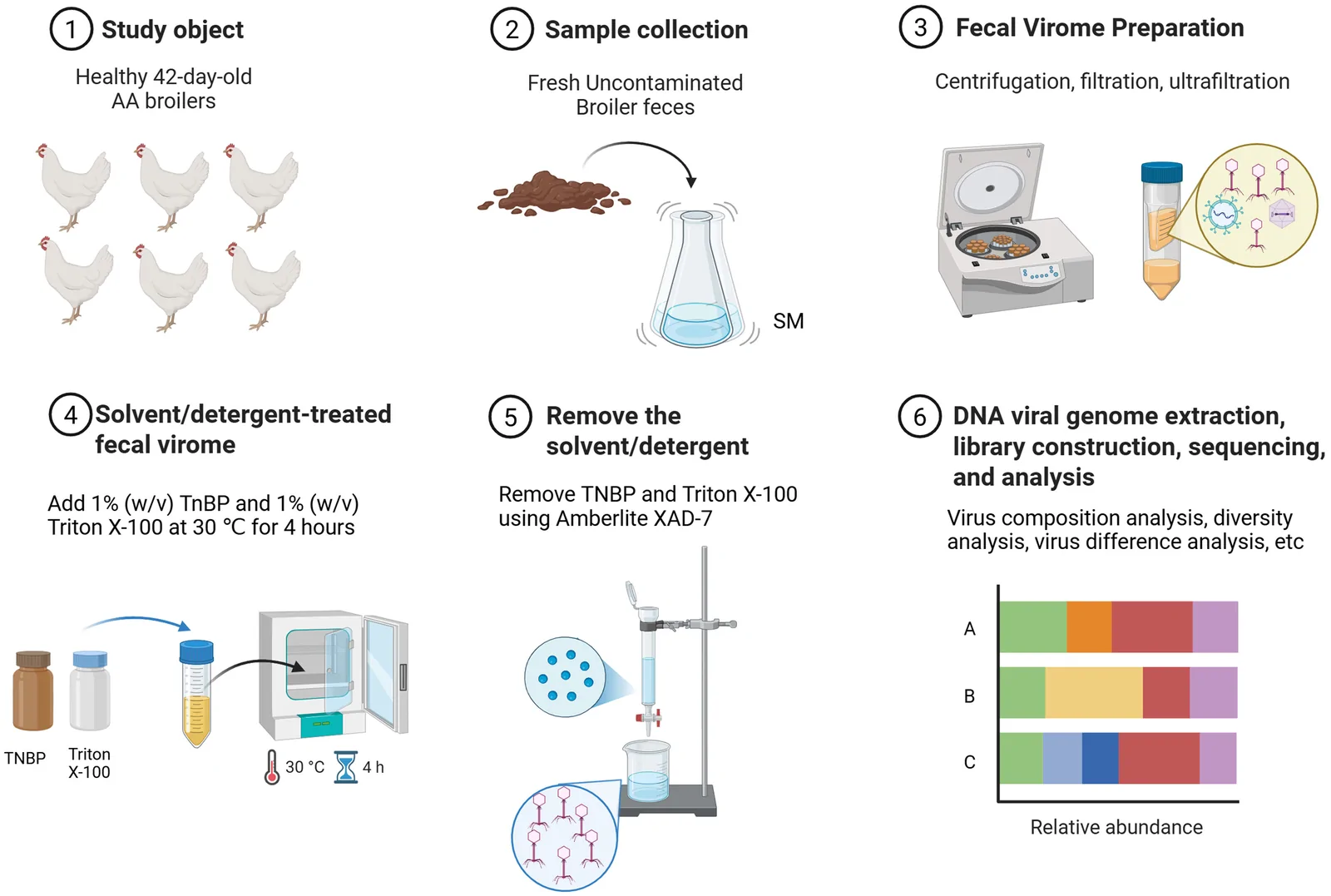
Preparation of FVT0 and FVT1 and sequencing analysis of DNA virome.

Quality control of raw sequencing data was performed using fastp (v0.23.4), retaining reads with a length of ≥ 75 bp as clean reads. Clean reads were aligned to the chicken reference genome (GCF_016699485.2) using Bowtie2 (v2.3.5.1), and host-derived reads were subsequently removed to obtain effective reads for downstream analysis. Effective reads from each sample were individually assembled into contigs of at least 1,000 bp in length using MEGAHIT (v1.2.9). Viral sequences were identified through an integrative approach employing four tools: VirSorter2 (v2.2.3; parameters: –min-length 1000, –min-score 0.9, –hallmark-required, –viral-gene-required), DeepVirFinder (v1.0; criteria: score ≥ 0.9, *p* < 0.01), VIBRANT (v1.2.1), and GenomeAD (v2.3.0; threshold: viral_score ≥ 0.7). Candidate viral sequences were defined as the union of results from these methods. The quality of predicted viral sequences was assessed using CheckV, yielding high-quality viral contigs per sample. Contig sequences representing both known and novel viruses across all samples were pooled and clustered into viral operational taxonomic units (vOTUs) based on the following thresholds: average nucleotide identity (ANI) ≥ 95% and coverage ≥ 85%. vOTU annotation followed the species classification framework outlined in ICTV MSL40.v1. A hierarchical integration of three complementary annotation strategies (in descending priority: 1 > 2 > 3) was applied: (1) Co-clustering of all vOTUs with complete reference genomes from ICTV MSL40.v1, InPhared, and RefSeq databases to infer species-level annotations based on shared cluster membership; (2) Family- or genus-level classification via vContact2-based viral cluster (VC) analysis incorporating both vOTUs and the InPhared database; (3) Annotation using VITAP (v1.7.1) with two customized databases—one integrating ICTV MSL40.v1, InPhared, RefSeq, and IMG/VR complete genomes, and another leveraging geNomad (v1.11.1, database version v1.9). Results from both database searches were harmonized by converting all taxonomic assignments to the ICTV MSL40.v1 standard and annotating vOTUs up to the family level. In cases of conflicting annotations between geNomad and VITAP, only non-conflicting hierarchical classifications were retained as final taxonomic assignments. Finally, effective reads were mapped back to vOTUs using Bowtie2 (v2.3.5.1) to quantify read counts and compute Reads Per Kilobase Million (RPKM) values, enabling the calculation of relative viral abundance at multiple taxonomic levels (family, genus, and species). We first performed α-diversity analysis using the phyloseq package (v1.42.0). This analysis aimed to calculate the Shannon and Simpson diversity indices, which are crucial indicators for reflecting the complexity and evenness of the viral community. To determine whether there were statistically significant differences in α-diversity between the two groups, Wilcoxon rank-sum tests were used, with a significance threshold of *p* < 0.05. Subsequently, β-diversity was evaluated via Principal Coordinate Analysis (PCoA) using phyloseq, and the intergroup distribution patterns were visualized in two-dimensional plots. To assess the statistical significance of group separation, Permutational Multivariate Analysis of Variance (PERMANOVA) was employed, also with a significance threshold of *p* < 0.05. In addition, to further compare viral composition between groups, violin plots were used to illustrate differences at the family level. For differential viral abundance analysis, we used DESeq2 (v1.38.0). The significance thresholds for this analysis were set as a log2 fold change ≥ 1 and a false discovery rate (FDR) < 0.05. Finally, we carried out protein functional annotations using HUMAnN3 (v3.1.1) with the option '–metaphlan - options "–add_viruses"'. After that, the results were standardized and quantified using the RPK (reads per kilobase) method. To present the results more intuitively, they were visualized as heatmaps Fig. 1.

## Results

### Taxonomic composition of the virome

**Fig. 2:**
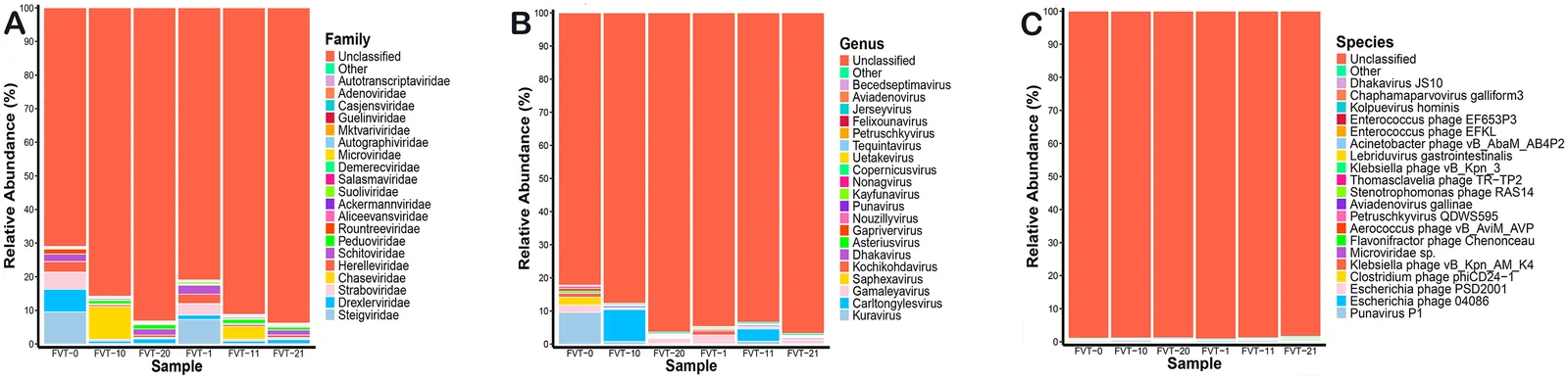
Relative abundance composition of viruses. (A) Family-level relative abundance composition of viruses. (B) Genus-level relative abundance composition of viruses. (C) Species-level relative abundance composition of viruses.

The vOTU sequences were annotated, identifying 1 kingdom, 7 phyla, 9 classes, 9 orders, 32 families, 127 genera, and 263 species. At the family level (Fig. 2A), the virome is predominantly composed of *Steigviridae, Drexlerviridae, Straboviridae, Chaseviridae, Herelleviridae, Schitoviridae*, and *Peduoviridae*, all being phage families. Moving on to the genus level, *Kuravirus, Carltongylesvirus, Gamaleyavirus, Saphexavirus, Kochikohdavirus, Dhakavirus, Asteriusvirus, Gaprivervirus, Nouzillyvirus* and, *Punavirus* are the primary components of the virome (Fig. 2B). Further analysis revealed that, at a more detailed classification level, including *Punavirus* P1, *Escherichia* phage 04086, *Escherichia* phage PSD2001, *Clostridium* phage phicD24-1, *Klebsiella* phage vB_Kpn_AM_K4, *Microviridae* sp, *Flavonifractor* phage Chenonceau and *Aerococcus* phage vB_AviM_AVP, were found to be relatively abundant (Fig. 2C).

### Analysis of α-diversity and β-diversity

**Fig. 3:**
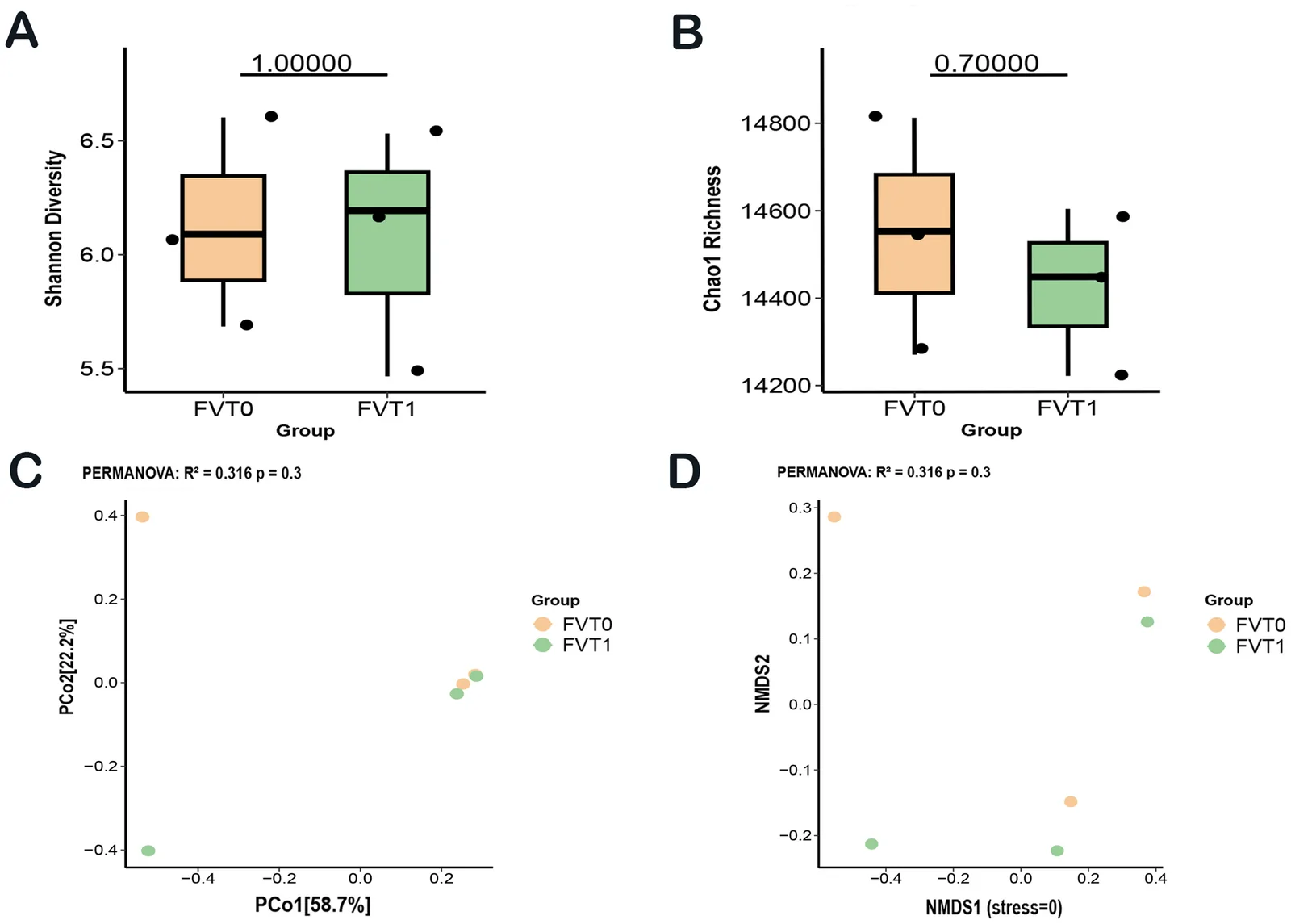
Analysis of α-diversity and β-diversity in virus composition (A) Shannon index, (B) Chao1 index, (C) Principal coordinate analysis (PCoA), (D)Non-metric multidimensional scaling analysis (NMDS).

We analyzed α-diversity and β-diversity to compare viral communities between FVT0 and FVT1. For the α-diversity analysis, as illustrated in Fig. 3A, the Shannon index was used to assess species richness and evenness in FVT0 and FVT1. As with the Shannon index results, no significant difference in species dominance was observed between FVT0 and FVT1 (*p* > 0.05). In addition, the Chao1 index was used to estimate the total number of true species in the sample. The results showed that the Chao1 index for FVT0 was slightly higher than that for FVT1, however, this difference was not statistically significant (*p* > 0.05; Fig. 3B). Regarding β-diversity analysis, principal coordinate analysis (PCoA) and non-metric multidimensional scaling (NMDS) were performed. As presented in Fig. 3C (PCoA) and Fig. 3D (NMDS), these analyses revealed a slight compositional difference between FVT0 and FVT1. Nevertheless, this difference was also not statistically significant (*p* > 0.05).

### Analysis of differences in virus abundance

**Fig. 4:**
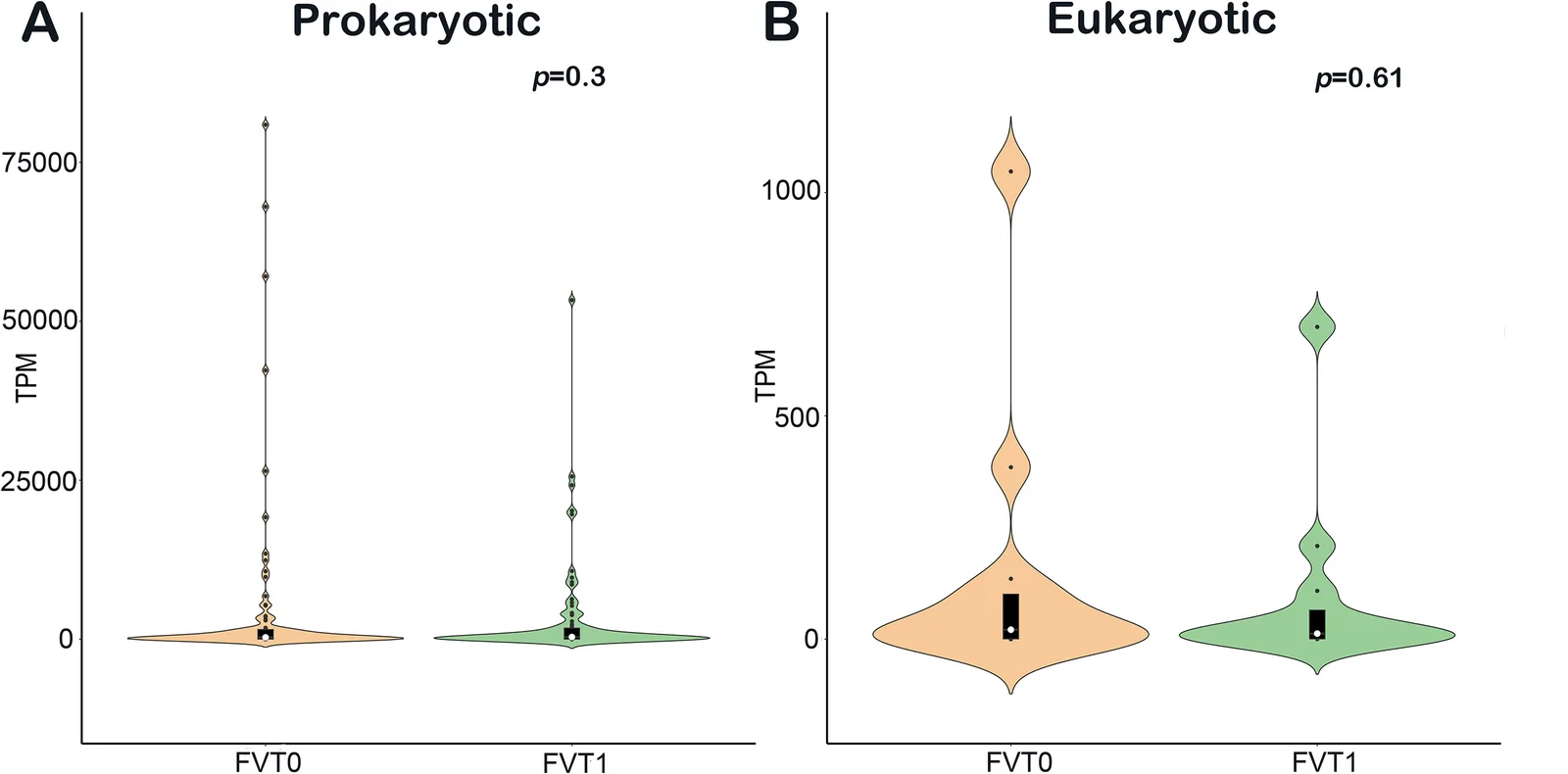
Analysis of the differences between eukaryotic viruses and prokaryotic viruses. (A) Differences in prokaryotic viruses. (B) Differences in eukaryotic viruses.

**Fig. 5:**
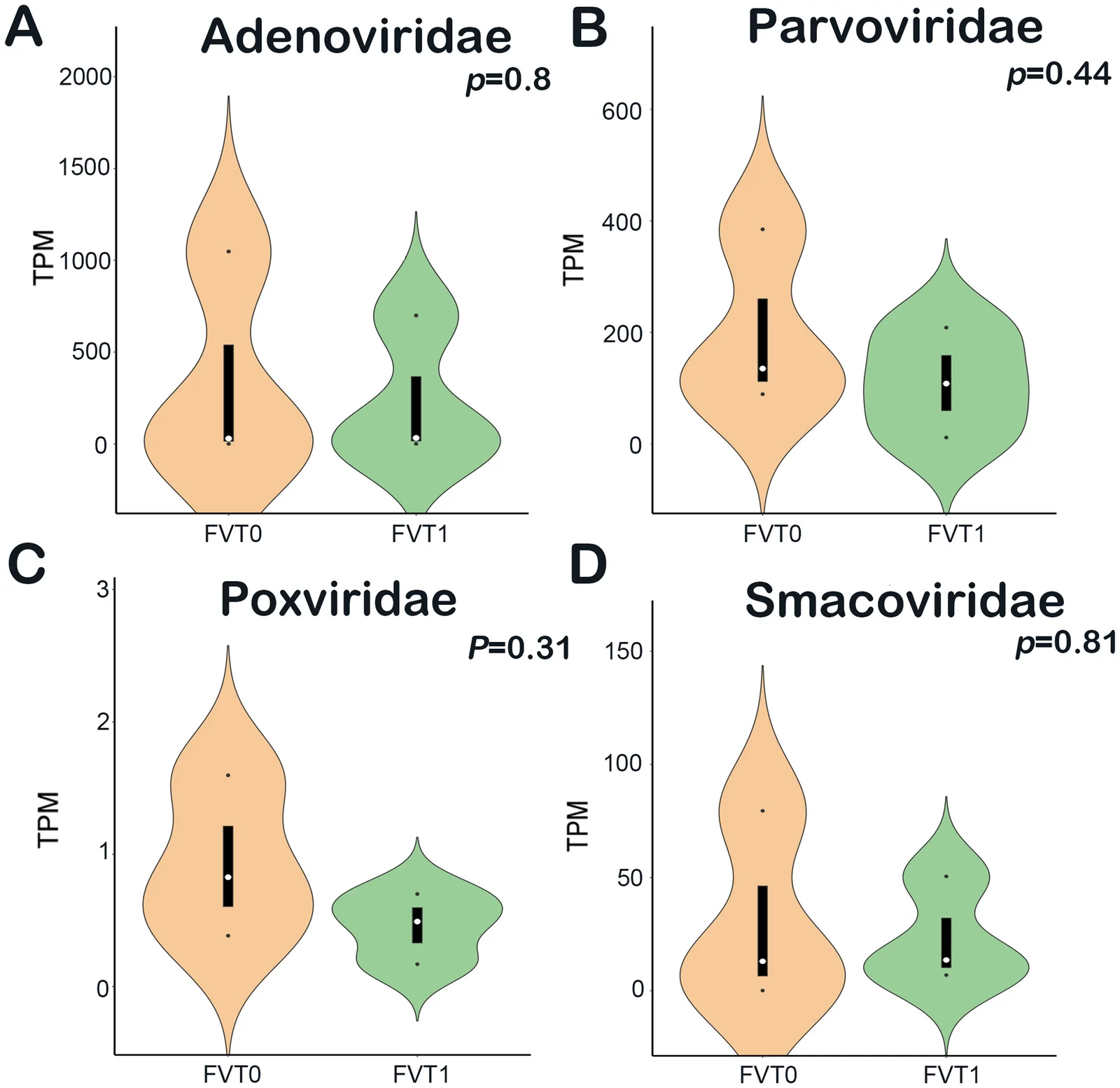
Relative abundance differences between families of eukaryotic viruses. A to D are respectively *Adenoviridae, Parvoviride, Poxviridae* and *Smacoviridae* of the eukaryotic virus families.

**Fig. 6:**
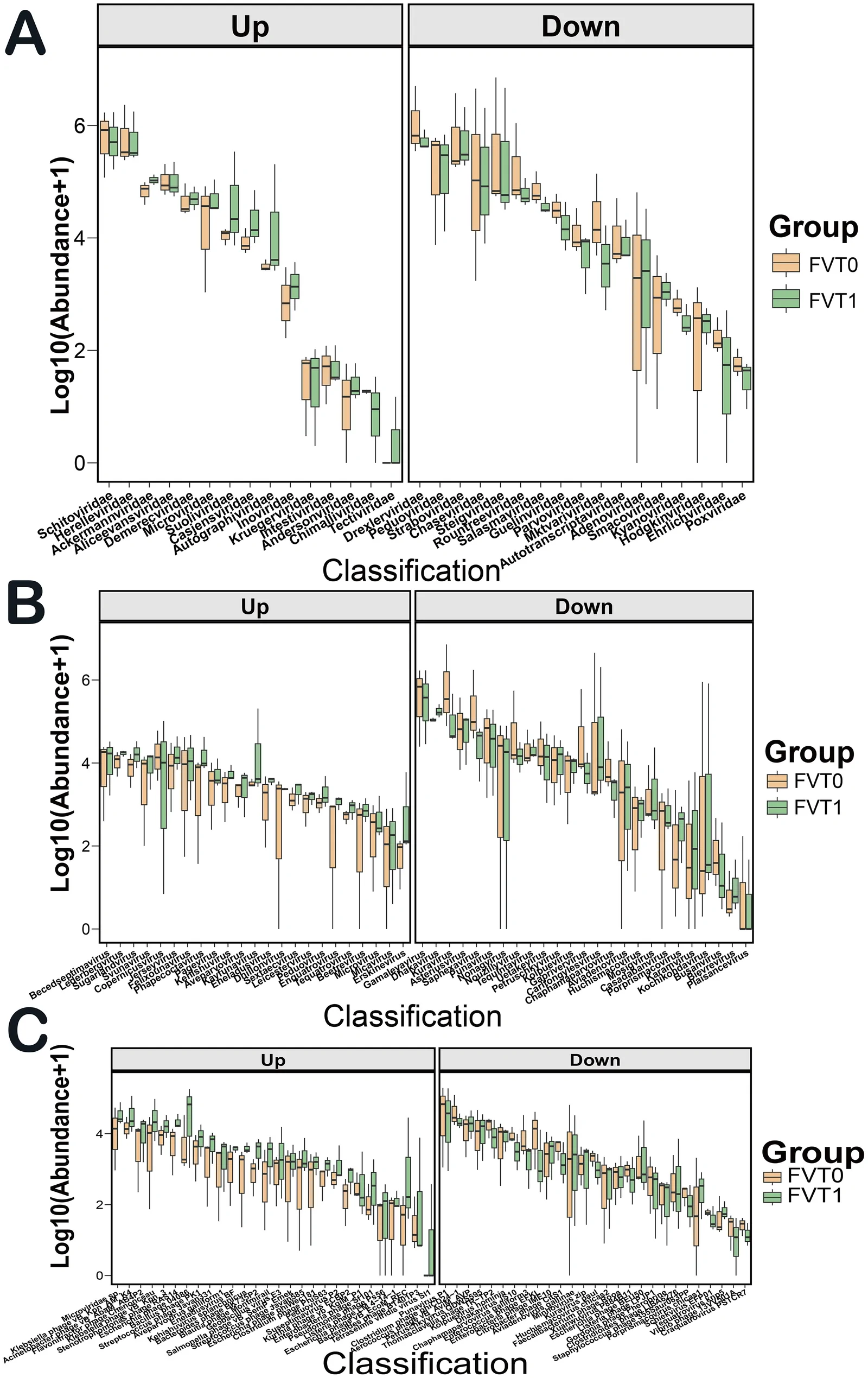
Analysis of virus differences. (A) Analysis of virus differences at the family level, (B) Analysis of virus differences at the genus level, (C) Analysis of virus differences at the species level.

As shown in Fig. 4, the relative abundance of Prokaryotic viruses showed no significant change between FVT0 and FVT1 (*p* > 0.05). In contrast, the abundance of Eukaryotic viruses trended to be lower in FVT1, although this difference was not statistically significant (*p* > 0.05). Analysis at the family level indicated that all four eukaryotic virus families decreased in FVT1 relative to FVT0, in the following order: *Adenoviridae, Parvoviridae, Poxviridae, Smacoviridae* (*p* > 0.05; Fig. 5). In addition, the relative abundance of several phage families, including *Schitoviridae, Herelleviridae, Ackermannviridae, Aliceevansviridae, Demerecviridae* and *Microviridae,* was significantly higher in FVT1 than in FVT0 (*p* < 0.05; Fig. 6A). Further analysis at the genus level revealed a significant increase in the relative abundance of phage genera, including *Becedseptimavirus, Lederbergvirus, Sugarlandvirus, Svunavirus, Copernicusvirus* and *Jerseyvirus* in FVT1 (*p* < 0.05). Only a small number of viral genera exhibited reduced relative abundance (Fig. 6B). At the species level, most of the identified viruses were common gut phages. Examples include *Microviridae* sp, *Klebsiella* phage vB_Kpn_AM_K4, *Flavonifractor* phage Chenonceau, *Escherichia* phage 04086, *Bacillus* phage PK1 W, *Eneladusvirus* BF, and *Salmonella* phage vB_SemP_Emek, all of which showed significantly increased relative abundance in FVT1 compared to FVT0 (*p* < 0.05). Conversely, a few phages, such as *Punavirus* P1, *Clostridium* phage phiCD24-1 and *Aerococcus* phage vB_AviM_AVP, were observed to be significantly decreased (*p* < 0.05; Fig. 6C).

### Differences in gene ontology (GO) and KEGG orthology (KO) functional annotations

**Fig. 7:**
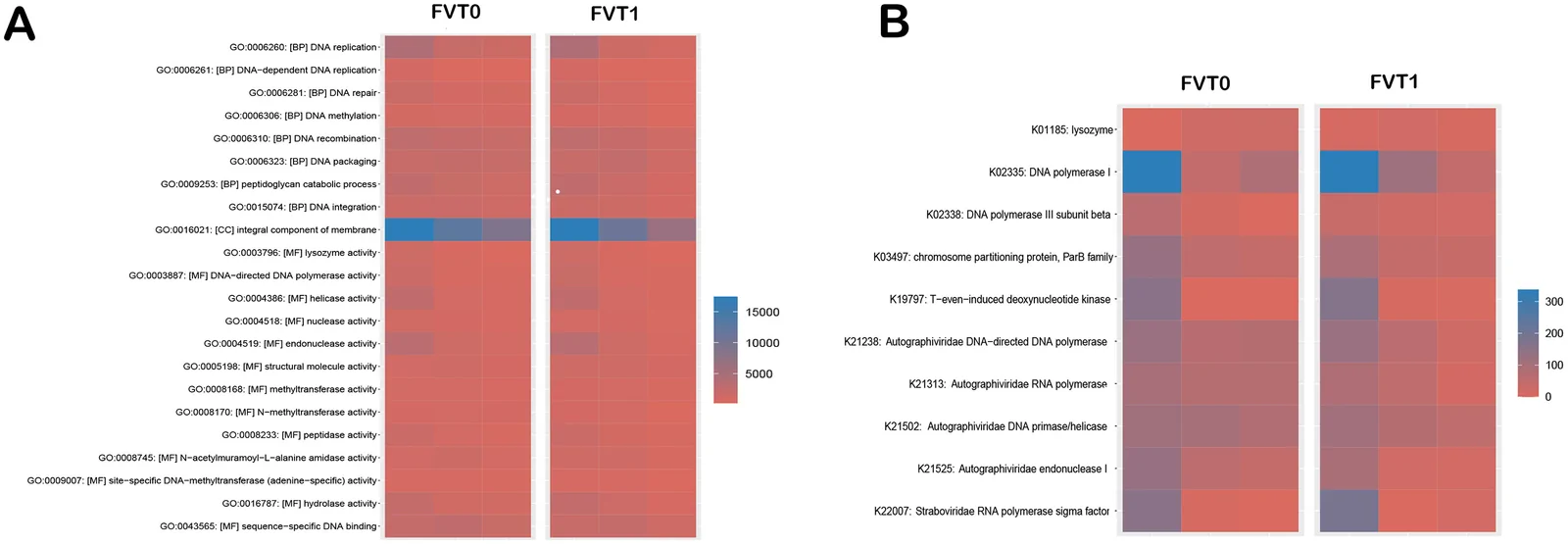
Analysis of virus function. (A) Gene ontology (GO), (B) KEGG orthology (KO).

Gene Ontology (GO) analysis divides functional terms into three main domains: Molecular Function (MF), Biological Process (BP), and Cellular Component (CC), as shown in Fig. 7A. The key difference in GO between FVT0 and FVT1 is the weaker response to eukaryotic viruses in FVT1. In the MF domain, 13 enzymatic activities were identified, including lysozyme (for bacterial cell wall breakdown), DNA polymerase (for DNA synthesis), helicase (for DNA unwinding), nuclease, and endonuclease activities. The BP domain includes eight processes. DNA replication and repair are vital for maintaining genetic material. DNA methylation regulates gene expression epigenetically. DNA recombination generates genetic diversity, and the peptidoglycan catabolic process is involved in bacterial cell wall degradation. Under the CC domain, "integral component of membrane" was annotated, indicating membrane-associated proteins or enzymes. Fig. 7B reveals differences in KEGG Orthology (KO)-associated enzymes and proteins, with FVT0 having a higher abundance. Viruses encode vital enzymes (e.g., DNA polymerase I for DNA synthesis/repair, T - even - induced deoxynucleotide kinase) and proteins (e.g., chromosome partitioning protein for viral genome distribution, *Straboviridae* RNA polymerase sigma factor) for replication, transcription, and nucleotide metabolism.

## Discussion

The gut microbiota plays an indispensable role in maintaining and regulating the host's metabolism and health. It encompasses a diverse array of microorganisms, including bacteria, archaea, fungi, and viruses, with phages being a particularly significant viral component (Wu et al., 2023). Phages, as prokaryotic viruses capable of infecting bacteria, are the predominant members of the gut viral community, and their abundance throughout the body, encompassing the intestine, mouth, and skin, far surpasses that of eukaryotic viruses (Keen and Dantas, 2018). Research has demonstrated that phages play a pivotal role in shaping the composition and function of bacterial communities. For instance, Hsu et al., 2019 found that phages affect the composition and function of bacterial communities and help maintain intestinal homeostasis by increasing functional diversity within bacterial or phage communities. Some eukaryotic viruses in the intestinal tract may cause infection or remain latent, while others undergo benign colonization and are unrelated to specific diseases. Some eukaryotic viruses, such as norovirus, rotavirus, and astrovirus, have been confirmed to be directly associated with diarrhea (Fulci et al., 2021). Given these observations, it is imperative to develop technical strategies to mitigate the risks posed by eukaryotic viruses in fecal virus transplantation (FVT) while preserving the biological activity of the majority of phages. Such an approach would offer effective safety guarantees for clinical applications.

In the present study, S/D (solvent/detergent) treatment was applied to FVT to selectively inactivate enveloped eukaryotic viruses. In the design and safety risk assessment of this process, representative enveloped pathogenic viruses specific to poultry, including avian influenza virus, Newcastle disease virus and infectious bursal disease virus, were fully taken into account. Even though these pathogens are theoretically undetectable in fecal samples obtained from healthy broilers, they were still classified as potential biological hazards threatening the biosafety of FVT. Subsequent DNA virome sequencing was conducted to validate the efficacy of this treatment. At the family level, the sequencing results revealed that the predominant viral families were phage-associated taxa. Further in-depth analysis at the genus and species levels confirmed a relatively high abundance of phages, which is consistent with previous findings indicating that phages dominate the FVT virome (Yu et al., 2023). α-diversity analysis, which assesses the richness and evenness of viral communities, showed no significant differences between samples (*p* > 0.05). β-diversity analysis, on the other hand, which examines the compositional differences between communities, suggested some variation between FVT0 (untreated FVT) and FVT1 (S/D-treated FVT), although these differences were not statistically significant (*p* > 0.05). However, targeted analysis of viral composition revealed a significant increase in the relative abundance of phages in FVT1 (*p* < 0.05), predominantly comprising common intestinal phages. This finding supports the potential application of FVT in treating intestinal disorders in chicks. At the family level, a slight reduction in the relative abundance of certain eukaryotic virus families, such as *Baculoviridae, Nudiviridae*, and *Adenoviridae*, was observed in FVT1 compared to FVT0. This observation suggests that S/D treatment tends to lower the relative abundance of eukaryotic viruses in FVT, although the difference was not statistically significant. Nevertheless, a minor decrease in the abundance of certain phages was also detected. This could potentially be attributed to interactions between the surface functional groups of the adsorption resin used in the S/D treatment and specific phages, leading to incomplete elution and reduced recovery efficiency (Lothert et al., 2020). Functional profiling of the virome was also conducted to gain insights into the biological activities of the viruses present. Gene Ontology (GO) analysis revealed enrichment in biological processes related to DNA replication, repair, methylation, recombination, packaging, and peptidoglycan degradation. These processes are characteristic of phage life cycles and suggest active involvement of viruses in nucleic acid metabolism and host cell wall remodeling (Raeisi et al., 2023). Additionally, GO terms encompassed various enzymatic activities, including lysozyme, DNA polymerase, endonuclease, methyltransferase, kinase, and peptidase activity. These activities indicate potential roles of the viruses in catalysis, post-translational modifications, and structural support during infection. KO (Kyoto Encyclopedia of Genes and Genomes Orthology) analysis further identified key viral-encoded enzymes and proteins. These included core components of DNA replication machinery, such as DNA polymerase I and III subunits, as well as deoxynucleotide kinases, DNA helicases, and endonucleases. The KO analysis findings corroborated the molecular functional annotations derived from GO analysis, providing a comprehensive understanding of viral functions.

FVT has demonstrated positive effects in the clinical treatment of various animal diseases. For example, Borin et al., 2023 found that FVT can influence the phenotypes of lean and obese mice by altering their gut microbiota. Rasmussen et al., 2023 reported that FVT enhanced the proliferation of *Akkermansia muciniphila* in the intestine and unexpectedly increased the fertility rate of laboratory mice. In broilers, Feng et al., 2024 demonstrated that FVT modulates intestinal microbiota structure in a milder way and can enhance the expression of tight junction proteins. Wu et al., 2024 found that both FMT and FVT could promote the inflammatory recovery of intestinal injury in broilers induced by lipopolysaccharide (LPS). Compared with FMT, FVT had less adverse stimulation on broilers and could reduce the transmission risk of pathogenic genes. With the global escalation of bacterial drug resistance, phage therapy has emerged as a promising alternative to antibiotics in the treatment of bacterial diseases. However, a single phage may sometimes fail to achieve the desired clinical effects. In contrast, FVT, which contains a collection of more than 90% phages, represents an effective alternative treatment for bacterial diseases (Raeisi et al., 2023).

Eukaryotic viruses in FVT can pose significant challenges as they may trigger immune responses in the host and even cause diseases. Although eukaryotic viruses are relatively fewer in number compared to phages, they are more effective in stimulating host immune responses (Seo and Kweon, 2019). Therefore, the method for removing eukaryotic viruses established in this study is important. Mao et al., 2024 demonstrated that FVT treated with S/D was effective in treating *Clostridium difficile* infection in mice. Compared with FMT and untreated FVT, the S/D-treated FVT reduced the mortality rate of mice. Among the 8 mice that received treatment, 7 showed negative results for *Clostridium difficile* in their feces as detected by qPCR. This study provides a methodological basis for the application of FVT in the treatment of animal diseases and also offers an effective reference for the FVT of white-feathered broilers prepared in this study to treat Salmonella infection in chicks.

Admittedly, the present study has several inherent limitations that should be acknowledged. First, we only conducted DNA virome sequencing, which quantifies the relative abundance of viral nucleic acids rather than directly reflecting the functional infectivity of intact viral particles. Although S/D treatment inactivates enveloped viruses by disrupting their lipid envelopes, it may not fully degrade viral genomic DNA, which could lead to overestimation of residual viral loads at the nucleic acid level. Second, the current investigation exclusively characterized DNA viruses while excluding RNA viruses from analysis. Accordingly, further metatranscriptomic profiling or RNA virome sequencing is warranted to comprehensively evaluate the decontamination efficiency of S/D treatment against diverse viral taxa. Third, rigorous *in vivo* validation using broiler animal models remains essential to confirm the practical biosafety of S/D-processed FVT under real production conditions, which will be prioritized in our follow-up studies. Notwithstanding these limitations, our findings provide preliminary mechanistic evidence that S/D treatment remodels the viral community composition of FVT, representing a promising and feasible strategy to improve its biosafety for poultry industry applications.

## Conclusions

In this study, the chicken fecal virome was treated with S/D, which tended to reduce the relative abundance of eukaryotic viruses while maintaining the activity and diversity of phages. This method may improve the safety of fecal virome transplantation, providing a theoretical basis and methodological support for its clinical application in the treatment of animal disease.

## CRediT authorship contribution statement

**Huimin Li:** Writing – original draft, Methodology, Investigation, Formal analysis, Data curation, Conceptualization. **Colin Buttimer:** Writing – review & editing, Visualization, Validation, Supervision, Project administration, Methodology, Formal analysis, Data curation. **Yujuan Zeng:** Methodology, Formal analysis, Data curation. **Xinglong Li:** Methodology, Investigation, Formal analysis, Data curation. **Yimin Jia:** Validation, Supervision, Investigation, Formal analysis. **Maoda Pang:** Visualization, Validation, Supervision, Funding acquisition. **Yue Li:** Project administration, Methodology, Investigation. **Hui Zhang:** Supervision, Project administration, Funding acquisition. **Yan Zhou:** Supervision, Methodology, Investigation. **Ran Wang:** Visualization, Validation, Supervision. **Hongduo Bao:** Writing – review & editing, Visualization, Validation, Supervision, Software, Resources, Project administration, Methodology, Investigation, Funding acquisition, Formal analysis, Data curation, Conceptualization.

## Disclosures

The authors declare that there is no conflict of interest.
